# More Efficient Ways of Assessing Treatments for Neglected Tropical Diseases Are Required: Innovative Study Designs, New Endpoints, and Markers of Effects

**DOI:** 10.1371/journal.pntd.0001545

**Published:** 2012-05-29

**Authors:** Piero Olliaro, Michel T. Vaillant, Shyam Sundar, Manica Balasegaram

**Affiliations:** 1 UNICEF/UNDP/World Bank/WHO Special Programme on Research & Training in Tropical Diseases (TDR), Geneva, Switzerland; 2 Centre for Tropical Medicine, University of Oxford, Oxford, United Kingdom; 3 Methodology and Statistical Unit, Centre for Health Studies, CRP-Santé, Luxembourg; 4 Institute of Medical Sciences, Banaras Hindu University, Varanasi, India; 5 Drugs for Neglected Diseases initiative (DNDi), Geneva, Switzerland; McGill University, Canada

## The Issue: Getting an Answer Sooner and Cheaper

Shortening trial time means reaching a decision earlier as to whether a treatment is effective—and saving money in the process. With old, invasive, inefficient tests of cure like those we have now for several neglected tropical diseases, follow-up (and total trial) times remain inefficiently and uneconomically long. While the need to licence new drugs is urgent for many of the neglected tropical diseases, it frequently takes 8–10 years from Phase 1 to licensure, and sometimes even longer. Consider visceral leishmaniasis: it has taken nearly 20 years and at least three different organisations for paromomycin to find its way to registration in India, and even longer to take a final decision to terminate the development of sitamaquine.

The need for efficiency is particularly acute in drug development, and specifically in Phase 2 clinical trials, when one will select the drug dose/schedule to be tested at a larger scale in the Phase 3 (pivotal) trials. Here, one wants to find out what works and what doesn't as quickly and economically as possible. Transposing results from non-clinical studies (in vitro and in vivo experiments) in terms of pharmacokinetic/dynamic correlation is not easy, so one is often left with a variety of potential doses and regimens to choose from.

## What Would Alternative Study Designs Add?

Adaptive trials designs are increasingly used by pharmaceutical companies to improve efficiencies in the R&D process [1]. This approach allows the possibility to redesign the trial (sample size, number of arms, etc.) based on the information acquired through interim analyses. Sequential and group sequential [1] trials are a special case of adaptive trials whereby several interim analyses are done in order to complete the trial earlier (interrupt enrolment) based on the accumulated information.

However, these methods work best for diseases for which treatment response becomes obvious shortly after treatment rather than having to wait for 6 months (visceral leishmaniasis), 18 months (onchocerciasis [river blindness]; human African trypanosomiasis [HAT; sleeping sickness]), or a yet-to-be-defined period for chronic Chagas disease. Tuberculosis is in the same league (18 months from treatment start for the initial assessment and another 12 months for final cure), while with “only” 28–63 days of follow-up, malaria is comparatively much better in this sense. The reason for such long follow-up times is that patients who initially respond favourably may relapse later, and such cases cannot yet be predicted by the current tests of cure.

There are several ways to specify early termination procedures (for futility), allow repeated analyses to be performed on accumulated data, maintain pre-specified α and β error, or stop the trial as soon as the information is sufficient to reach a conclusion [2]. These methods can be grouped as: (i) sequential methods (sequential probability ratio test and triangular test [2], [3]) and (ii) group sequential designs (Peto [4], Pocock [5], and O'Brien-Fleming [6] methods; α [7], [8] and β [9] spending function; etc.). This is a domain of ongoing statistical research with existing methods being improved and new ones developed.

## Example: Triangular Test for Visceral Leishmaniasis

We used a triangular design to study different doses and durations of combination treatments for visceral leishmaniasis in India [10]. Experimental studies had been inconclusive [11] while toxicology studies had shown the combinations to be safe (preclinical toxicology studies on several drug combinations have been done, with no major safety concerns identified [Drugs for Neglected Diseases initiative (DNDi), data on file]).

**Figure 1:**
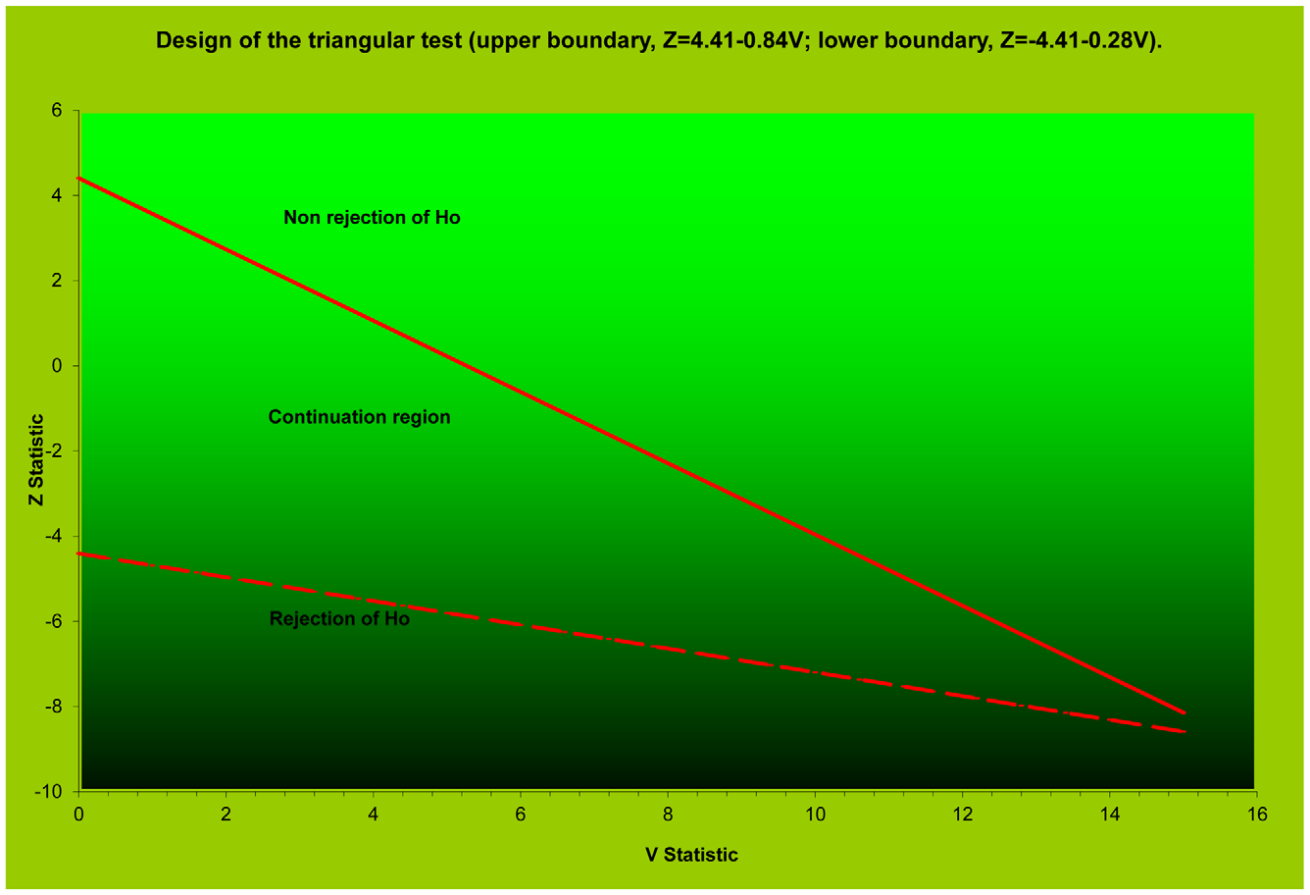
Design of the triangular test for a Phase 2 study of anti-leishmania drug combinations.

The trial was designed as a randomized, parallel-arm, non-comparative, open-label study using the group-sequential triangular test method to reach, with the minimum number of subjects, an early decision as to which of four regimens should be selected for additional testing. With a type 1 error α = 5% and power 1−β = 95% assumptions, considering a failure rate <10% as adequate efficacy (the minimum detectable failure rate at the β = 5% level) and a failure rate ≥25% as insufficient efficacy, the boundaries of the test were calculated for H0 (p = p0) and Ha (p<pa) with p0 = 0.25 and pa = 0.10. Based on simulations, we expected the sample path to cross the H0 rejection line with an average sample size of 40 patients and the H0 non-rejection line with an average sample size between 20 and 25 patients. When, after enrolling 45–46 patients per arm, all treatments appeared equally and highly effective, an additional 45 consecutive patients were enrolled and non-randomly assigned to a fifth regimen (Figure 1).

**Figure 2:**
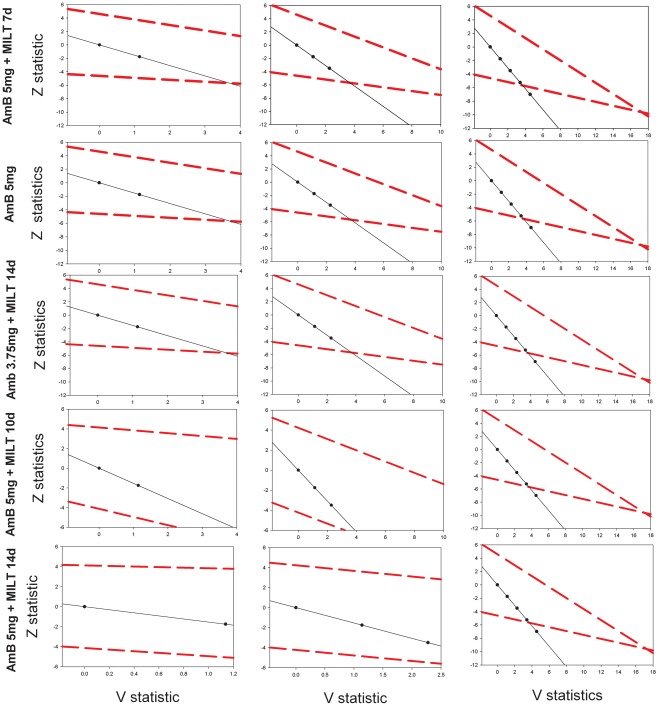
Patient enrolment in a Phase 2 study of visceral leishmaniasis with triangular design.

All 181 subjects in Groups A–D completed assigned the treatment, and on day 16, 100% showed parasite-free splenic aspirate smears and fulfilled the criteria for apparent cure (Figure 2). Following the successful completion of this study in India, DNDi used this design again in a Phase 2 trial of anti-leishmania drug combinations in Africa (ClinicalTrials.gov NCT01067443).

Using this approach did not result in shortening trial time; the maximum calculated number of patients was to be enrolled as all treatment regimens proved very effective. However, an economy was achieved in the number of trial subjects and the time to reach a conclusion. Though the two methods cannot be strictly compared, a classical single-stage comparative trial design, with a type 1 error α = 5% and a power 1−β = 95% and the null hypothesis of 90% efficacy, would require a sample size of 580 patients per arm to reach the significance level for a regimen with 95% efficacy. The two approaches test different hypotheses, but, especially for dose-finding purposes, the triangular test offers clear advantages in screening different treatment regimens.

## Better Measures of Treatment Outcomes Are Needed to Make Adaptive Designs Worthier

The main indication for adaptive trial designs such as the triangular design will indeed be in the futility setting; weeding out ineffective experimental doses in Phase 2 and thus reducing the number of patients at risk of being exposed to ineffective doses. This will shorten time to decision and moderate expenses. It will be interesting to see how the triangular design performs in situations like African leishmaniasis (see above) where treatments tend to be comparatively less effective than in India, and thus arms could be dropped earlier. The triangular design however would probably be less useful in diseases like HAT for example, where end-of-treatment outcomes tend to be less informative.

However, until and unless reliable markers of treatment effects are found, clinical trials and drug development for neglected tropical diseases will be hampered. More investments are needed in this area. An expensive marker can be tolerated for drug development (contrary to patient management, which needs inexpensive, non-invasive tests) because the net result will be a curtailment of time and overall cost of development. However, such markers are notoriously difficult and expensive to discover and validate; attention must be called to this area for the required long-term investments to be made. Meanwhile, immediate solutions are also needed.

## Beyond Traditional Approaches

What can be done now and with limited resources?

Action must be taken to increase awareness of the problem among research funding organizations and the research community itself for novel solutions to be found and tested. Consideration should be given also to approaches used in different areas (such as non-transmissible diseases). It is hoped that this paper will stimulate interest and broaden the debate.

In leishmaniasis treatment trials, as for other diseases, an initial (apparent) cure can be followed by a relapse (or a re-infection, to complicate matters further), whereby the final cure rate will be lower than the initial one. So, a fundamental question is how predictive of final cure the initial response is. The answer may vary with the outcome, disease, treatment, parasite, and patient population, and thus location of trial. In the leishmaniasis triangular trial cited here, we used Day 16 for the decision based on initial cure and 9 months (instead of the customary 6 months) for final cure. As all the treatment regimens tested were highly effective, Day 16 proved to be a reliable indicator of success; the same would apply to the other extreme case of very ineffective treatments (in our study, it would have required about half as many patients). The problem will reside in treatments that are only partly effective, which will suppress parasite replication temporarily or kill the majority but not all the parasites; initially, these will be missed by insensitive diagnostics, only to rebound to be detected later on during follow-up.

To some extent, available tools may be fit for purpose. For example, with no cheaper tools in sight, trial sites could be provided with some state-of-the-art tools such as real-time (RT) PCR, which could predict cures or relapses based on the number of organisms at the end of treatment with reasonable accuracy [12]. However even RT-PCR needs to be validated and standardised for the respective diseases. Currently DNDi, Médecins Sans Frontières (MSF), the World Health Organization (WHO: Special Programme for Tropical Diseases [TDR], Pan-American Health Organization [PAHO]), and various researchers are working together towards validating the use of quantitative RT-PCR as treatment outcome measure in Chagas disease.

But there may be also other options involving imaginative, cost-effective ways of constructing the evidence base to design trials differently. In this paper we focus more on Phase 2-type trials, but the concept should be extended to larger pivotal trials and pragmatic trials as well.

Progress has been made with the design of tuberculosis and malaria treatment trials, which will specially benefit Phase 2. For tuberculosis, concern has been raised over the use of early-response methods such as (extended) early bactericidal activity [13] and serial sputum colony counts (SSCC) [12], [14] to predict efficacy, over shortened duration of follow-up (how informative are results at 6 months instead of 2 years [15]), and over more general design issues [16] and use of surrogate endpoints [17]. In malaria, too, research has been done on identifying both optimal duration of follow-up for establishing final response [18] and also early outcome measures (Day 3) which are predictive of parasite susceptibility [19].

Some of the examples above show that research question-driven collection and analyses of databases from previous trials are both useful and cost-effective as a means of developing newer, evidence-based approaches.

## In Summary

- Shortening trial time and reducing requirements for patients saves time and money, and spares patients from unnecessary exposure: there is therefore both an economic and an ethical motive for rationalizing trial design.
- Economies can be found with alternative clinical trial designs, such as adaptive trials (especially in the futility setting), though these are only partly suited for neglected tropical diseases, which have inadequate measures of treatment outcomes.
- Research is needed into generating better tests of treatment outcomes for neglected tropical diseases, but sizeable long-term investments are required.
- New, imaginative approaches should be investigated that will generate an evidence base for alternative trial designs.

## Abbreviations

DNDi: Drugs for Neglected Diseases initiative
HAT: human African trypanosomiasis
MSF: Médecins Sans Frontières
PAHO: WHO Pan-American Health Organization
RT-PCR: real-time PCR
SSCC: serial sputum colony counts
TDR: WHO Special Programme for Tropical Diseases
WHO: World Health Organization
